# Beyond overdiagnosis: reframing autism prevalence through a neurodivergent phenotype lens

**DOI:** 10.1016/j.jped.2025.101467

**Published:** 2025-11-13

**Authors:** Simone Hauck, Luciana Terra de Oliveira

**Affiliations:** a Universidade Federal do Rio Grande do Sul (UFRGS), Programa de Pós-Graduação em Psiquiatria e Ciências do Comportamento, Porto Alegre, RS, Brazil; b Hospital de Clínicas de Porto Alegre (HCPA), Laboratório de Psiquiatria Psicodinâmica, Porto Alegre, RS, Brazil

Dear Editor,

We read with great interest the review by Castro, Riesgo, and Gadia (2025) [1], which offers a valuable critical analysis of the factors influencing the rising prevalence of Autism Spectrum Disorder (ASD). The authors correctly highlight the role of methodological, clinical, and sociocultural determinants, and their synthesis is timely and important. We would, however, like to extend the discussion to a more fundamental issue: the conceptual framework that underlies current diagnostic systems.

The central concern, in our view, is that the categorical nosology on which ASD diagnosis relies may itself be driving both the perception of “overdiagnosis” and the persistence of underdiagnosis in certain groups. From a clinical perspective, what we observe is not multiple discrete disorders proliferating, but rather a single heterogeneous neurodivergent phenotype, variably expressed across individuals. Depending on which area of vulnerability (“desert”) dominates—for instance, language, attention, or social reciprocity — the same phenotype may be labeled as ASD, ADHD, or another condition. Conversely, areas of strength (“islands”) may obscure the underlying similarities across presentations.

This view suggests that the apparent “epidemic” of autism is, to a large extent, an artifact of how we draw diagnostic boundaries. The steep increase in prevalence rates may thus reflect not only earlier screening, improved awareness, or expanded criteria, but also the intrinsic limitations of categorical classification in capturing the complexity of neurodevelopmental variation [2,3].

Such concerns resonate with broader debates in psychiatry regarding the adequacy of categorical diagnoses as guides for research and treatment. The field of precision medicine has emphasized the need to move beyond rigid labels, arguing that psychiatric disorders are better understood through dimensional constructs that integrate genetic, neurobiological, and behavioral domains [4]. History also provides cautionary lessons: the rapid expansion of the diagnosis of pediatric bipolar disorder in the United States led to a sharp increase in the prescription of antipsychotics to children—a development now widely criticized as the consequence of diagnostic inflation [5,6]. These examples underscore how nosological choices directly shape epidemiological curves, clinical practice, and even patterns of pharmacological use.

Importantly, this challenge is not limited to childhood. Also, in adults with neurodivergent profiles, category-centered practice has often led to broad and imprecise treatment strategies. Recent analyses in the bipolar spectrum show that antipsychotic management should be guided by phenotypic markers — excitability, sleep patterns, metabolic vulnerability—rather than by the diagnostic label alone [7]. This convergence suggests that a phenotype-first orientation is useful across the lifespan, not only in pediatric populations.

Dimensional and integrative models of neurodivergence — which conceptualize developmental diversity as a spectrum of strengths and vulnerabilities — may offer a way forward. They could help reduce diagnostic inflation in borderline cases, avoid fragmentation of similar phenotypes into competing categories, and support more personalized and context-sensitive interventions. Importantly, this shift would not deny the need for accurate diagnosis to ensure access to services; rather, it would help safeguard the clinical and social meaning of such diagnoses by anchoring them in a broader understanding of neurodiversity.

In conclusion, while overdiagnosis is an important concern, the deeper challenge lies in whether the current classificatory model is adequate. We encourage the journal to foster further debate on frameworks that move beyond categorical labels toward a dimensional understanding of neurodivergent phenotypes.

Sincerely.

## Declaration of generative AI and AI-assisted technologies in the manuscript preparation process

During the preparation of this work, the authors used ChatGPT (OpenAI, San Francisco, CA, USA) to assist in language editing and formatting in accordance with journal guidelines. After using this tool, the authors reviewed and edited the content, and take full responsibility for the content of the published article.

## Funding

No specific funding was received for this work.

## Data availability

The data that support the findings of this study are available from the corresponding author.

## Conflicts of interest

The authors declare no conflict of interest.
